# Is the innovative both column screw fixation technique a biomechanical game-changer in the fixation of acetabular posterior column fractures?

**DOI:** 10.1007/s00264-025-06604-2

**Published:** 2025-07-19

**Authors:** Vedat Öztürk, Burak Kaya, Talip Çelik, Malik Çelik, Cemal Kural, Mustafa Gökhan Bilgili

**Affiliations:** 1 Bakırköy Dr.Sadi Konuk Eğitim ve Araştırma Hastanesi, Istanbul, Turkey; 2 Şanlıurfa Mehmet Akif İnan Eğitim ve Araştırma Hastanesi, Sanliurfa, Turkey; 3 Kocaeli University, İzmit, Turkey

**Keywords:** Acetabular fracture, Lag screw fixation, Both column screw, Magic screw, Posterior column screw

## Abstract

**Purpose:**

The Both Column Screw (BCS) fixation technique is a recently introduced, innovative method for the treatment of acetabular posterior column fractures. This study aims to biomechanically compare the BCS technique with conventional posterior column lag screw fixation methods using finite element analysis.

**Methods:**

Five different internal fixation models were simulated using five distinct screw fixation techniques: antegrade posterior column screw (APCS), retrograde posterior column screw (RPCS), magic screw (MS), anterior BCS (aBCS), and posterior BCS (pBCS). The modeling process included meshing, assignment of material properties, and definition of boundary conditions. Each model was subjected to three different loading conditions: level walking, stairs up, and stairs down. The biomechanical performance of each fixation technique was evaluated based on five parameters: maximum stress in the screw, maximum stress in the bone, total deformation, gap in fracture surfaces, and sliding distance in the fracture surface.

**Results:**

Finite element analysis demonstrated biomechanical differences among the five fixation techniques. The APCS model consistently showed the highest stress values and deformation across all loading conditions, whereas the MS, aBCS, and pBCS models exhibited lower deformation and stress parameters. Among these, pBCS generally displayed the most favorable performance in terms of stress reduction and fracture stability. Overall, the BCS configurations (aBCS and pBCS) showed improved biomechanical behavior compared to conventional fixation methods.

**Conclusion:**

The BCS fixation technique, due to its superior biomechanical properties, may serve as a valuable addition to current methods for acetabular posterior column fractures. It broadens surgical options and may support clinical decision-making for orthopaedic surgeons.

## Introduction

Acetabular fractures represent a complex and clinically significant group of injuries in orthopaedic trauma surgery, typically resulting from high-energy trauma in younger patients or low-energy falls in the elderly [1]. The intricate three-dimensional anatomy of the pelvis, proximity to critical neurovascular structures, and the necessity of restoring hip joint congruity make these fractures particularly challenging to manage [2, 3]. Contemporary epidemiological studies indicate that posterior column fractures—whether isolated or as part of associated patterns—account for approximately 10–25% of all acetabular fractures, with higher prevalence observed in complex fracture types [1, 4, 5].

Traditional management of displaced acetabular fractures involves open reduction and internal fixation (ORIF) using plates and/or lag screws [5, 6]. However, advancements in imaging and surgical instrumentation have spurred interest in minimally invasive techniques, which aim to reduce soft tissue damage, perioperative morbidity, and recovery time. Among these, percutaneous lag screw fixation has gained prominence due to its ability to achieve interfragmentary compression with limited exposure [7, 8].

Various lag screw techniques are available for posterior column fixation, including the antegrade posterior column screw (APCS), retrograde posterior column screw (RPCS), and the magic screw (MS) [9–11]. While APCS requires insertion through the lateral window of the ilioinguinal approach, RPCS and MS can be placed percutaneously under fluoroscopic guidance, offering the benefits of minimally invasive surgery [12, 13].

Recently, a novel osseous pathway termed the Both Column Fixation Corridor (BCFC) has been introduced for posterior column fixation [14]. This anatomical corridor traverses both the anterior and posterior columns, enabling the placement of a transiliac antegrade lag screw—the Both Column Screw (BCS)—which originates from the iliac crescent and extends toward the lesser sciatic notch. In their original description of the technique, Öztürk et al. demonstrated that two distinct lag screws can be inserted within this corridor—one positioned more anteriorly near the acetabular articular surface (aBCS), and the other posteriorly, close to the greater sciatic notch (pBCS), which provides multiple fixation options for posterior column fractures [14, 15]. Although the BCS technique shows promise for percutaneous fixation, its biomechanical performance remains uncharacterized, and no direct comparisons with conventional methods exist in the literature.

This study employs finite element analysis (FEA) to biomechanically compare five posterior column fixation strategies—APCS, RPCS, MS, anterior BCS (aBCS), and posterior BCS (pBCS)—under simulated physiological loads, evaluating stress distribution, deformation, and fracture gap behavior for each configuration, with aBCS and pBCS analyzed separately to assess their individual biomechanical profiles relative to conventional techniques.

## Materials and methods

### Model development

A three-dimensional model of a healthy human pelvis was generated using computed tomography (CT) images (slice thickness: 0.625 mm) obtained from a 37-year-old male individual (175 cm in height, 82 kg in weight, Body Mass Index: 26.8). The CT data were segmented using MIMICS 12.0 software (Materialise, Leuven, Belgium) to reconstruct the initial 3D anatomical structure. Surface irregularities and modeling artifacts, such as intersecting or discontinuous surfaces, were corrected using Geomagic Studio 10 (Raindrop Inc., USA). The finalized solid model was then exported in IGES format to SolidWorks (Dassault Systèmes, SolidWorks Corp., USA) for further modeling procedures.

Subsequently, a posterior column fracture of the left acetabulum was simulated on the model using SolidWorks, in accordance with the Judet and Letournel classification system [16].

**Fig. 1:**
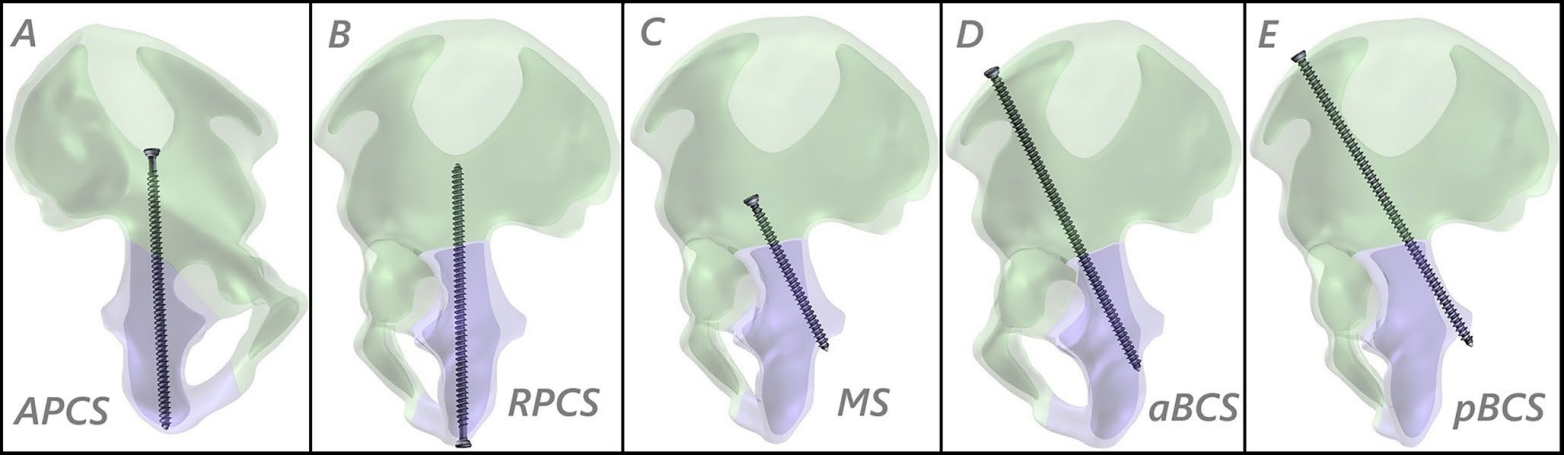
Schematic representations of the five screw fixation configurations evaluated in this study: antegrade posterior column screw (APCS), retrograde posterior column screw (RPCS), magic screw (MS), anterior Both Column Screw (aBCS), and posterior Both Column Screw (pBCS)

For internal fixation, 6.5 mm diameter titanium alloy screws—commonly used in pelvic and acetabular surgeries—were modeled. Five different screw fixation techniques were simulated: antegrade posterior column screw (APCS), retrograde posterior column screw (RPCS), Magic Screw (MS), anterior Both Column Screw (aBCS), and posterior Both Column Screw (pBCS), as illustrated in Fig. 1. All assembled models were saved in STEP format and imported into ANSYS Workbench (ANSYS Inc., Canonsburg, PA, USA) for finite element analysis (FEA).

### Material properties, meshing and contact assignments

The material properties of all models were assumed to be linear, elastic, and isotropic. Since Ti6Al4V is commonly used for acetabular fracture fixation screws, this material was assigned to the metal screws in the ANSYS Workbench software. The material properties of the screw model and those of the cortical and cancellous regions of the pelvic model were defined separately [17–23]. All assigned material properties are presented in Table 1.

**Table 1:** Material properties of pelvis cortical and cancellous models and metal screw models

Models	Elastic Modulus (MPa)	Poisson Ratio	Density (kg/m^3^)	Strength of the materials (MPa)	References
**Pelvis Cancellous Bone**	132	0.2	350	12	[16–18]
**Pelvis Cortical Bone**	17,000	0.34	1800	150	[19–21]
**Metal Screw**	114,000	0.314	4400	950	[22]

**Fig. 2:**
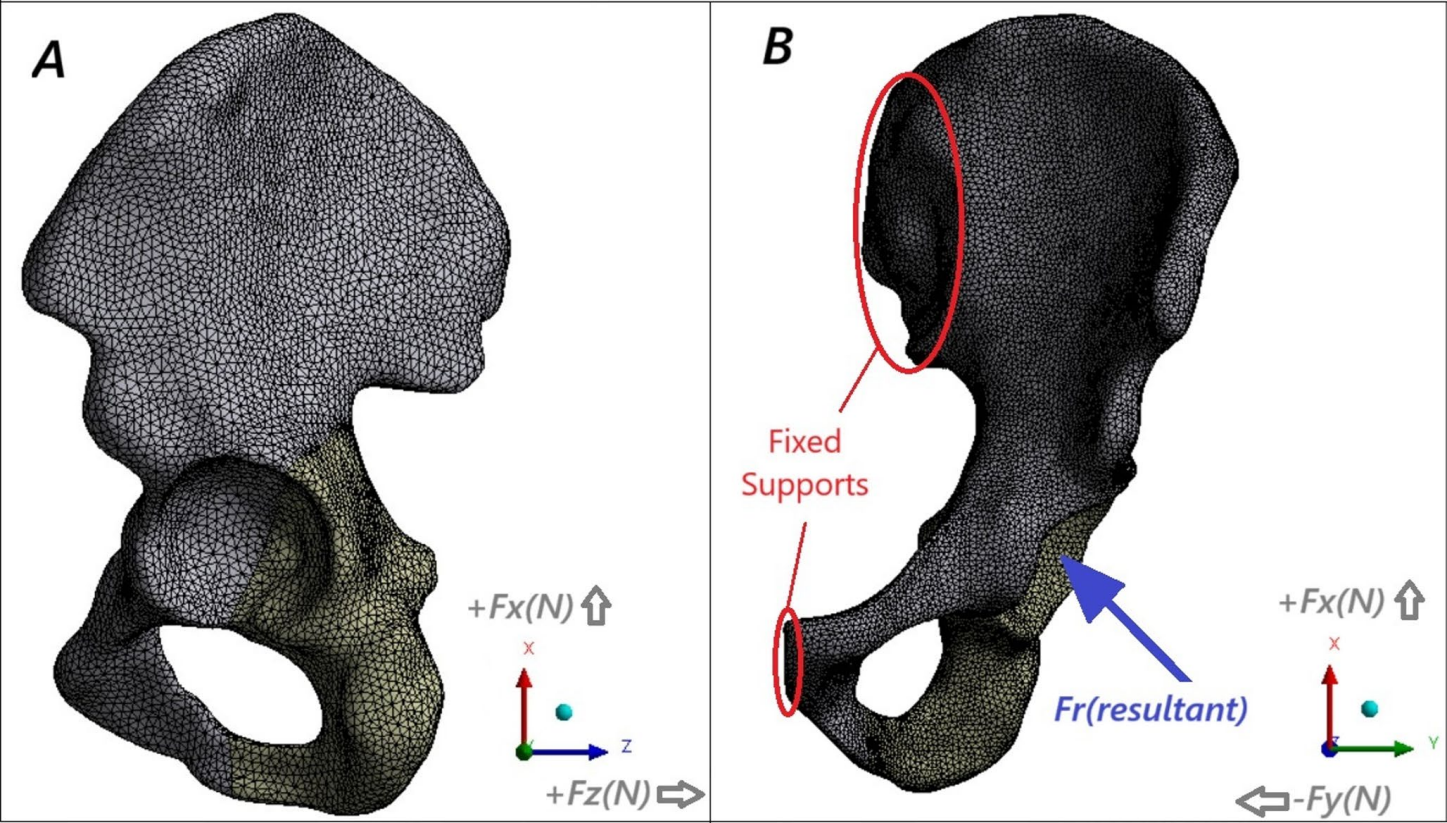
(**A**–**B**) Different views of the pelvic model according to the coordinate system. The FEA model of the pelvis is shown with the applied forces and boundary conditions. Force vectors are illustrated along the X, Y, and Z axes, and the resultant force vector (r) is also depicted. F: force; x, y, z: coordinate axes; r: resultant force

Mesh generation was performed following a mesh convergence study. Convergence was assessed by refining the element size from 1 mm to 0.2 mm in 0.1 mm intervals for the screw model, and from 6 mm to 1 mm in 1 mm intervals for the cortical and cancellous regions of the pelvis model. The most appropriate element sizes for optimal FEA results were determined to be 3 mm for the pelvis and 0.5 mm for the screw models. Additional refinements were applied to the contact regions to ensure convergence. A SOLID187 tetrahedral element type, consisting of 10 nodes with three degrees of freedom per node, was used in all finite element models. Following meshing, the models included approximately 730,000 nodes and 472,000 elements, as shown in Fig. 2.

Five different FEA models were generated and named according to the abbreviation of each fixation method. All contacts were defined as frictional, with a friction coefficient of 0.3 for screw–bone interfaces and 0.35 for fracture surface interfaces, as reported in the literature [24, 25].

### Load and boundary conditions

The load and boundary conditions applied to the pelvis–screw models are illustrated in Fig. 2. The models were subjected to physiological loads corresponding to three common daily activities: level walking, stairs up, and stairs down, in accordance with values reported in the literature for individuals walking at normal speed or ascending/descending stairs [26–29].

In accordance with these references, the peak resultant force acting on the acetabulum during each activity cycle was determined. The orthogonal components of this resultant force along the X, Y, and Z axes were then applied simultaneously to the models, subjecting them to loading in all three axes. During loading, the pelvis models were fixed at the sacroiliac joint and pubic symphysis.

The specific load magnitudes for each activity, including the orthogonal force components along the X, Y, and Z axes and their corresponding resultant vectors, are summarized in Table 2.

**Table 2:** Loads values in accordance with the activity

Activity	Fx (*N*)	Fy (*N*)	Fz (*N*)	Fr (*N*)	References
**Level walking**	611,16	-605,13	2114,48	2282,7	[25, 26]
**Stairs up**	440,44	-476,4	1774,0	1888,9	[25, 27]
**Stairs down**	679,61	-333,75	2161,11	2289,9	[25, 28]

## Result

**Fig. 3:**
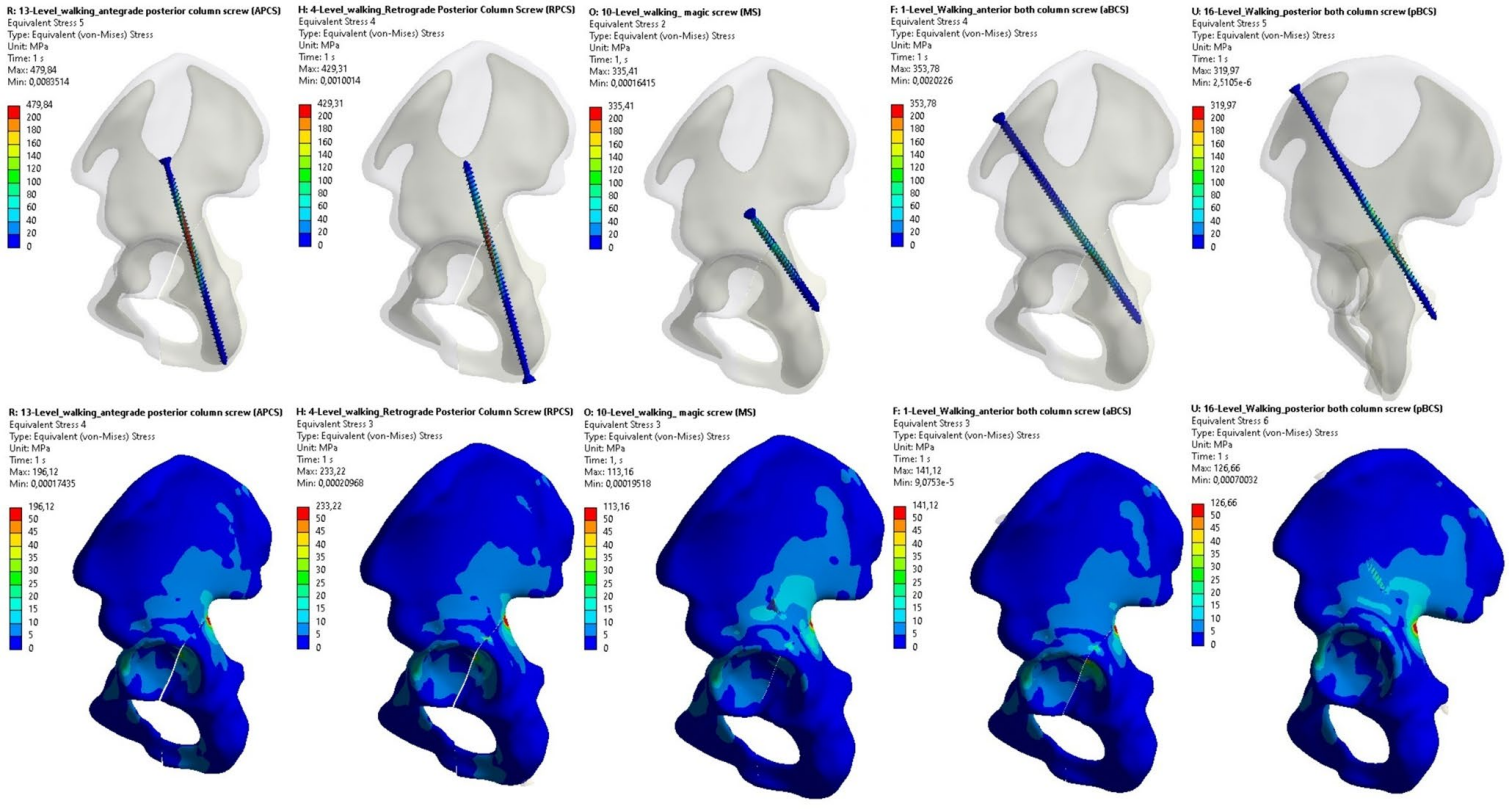
Representative von Mises stress distributions in both screws and surrounding bone during the level walking condition for each fixation model: APCS, RPCS, MS, aBCS, and pBCS

The findings of the finite element analysis are summarized in Table 3, which presents the maximum von Mises stress values in both the screws and the surrounding bone, as well as total deformation measurements, for five different internal fixation models under three simulated physiological loading conditions: level walking, stairs up, and stairs down. As a representative example, the von Mises stress distributions for both bone and screw components during the level walking scenario are visually presented for all fixation models in Fig. 3.

**Table 3:** Stress and deformation metrics for different screw fixation methods

	Level Walking			Stairs Up			Stairs Down		
Screw Type	Max Stress in Screw (von Mises) MPa	Max Stress in Bone (von Mises) MPa	Total Deformation (mm)	Max Stress in Screw (von Mises) MPa	Max Stress in Bone (von Mises) MPa	Total Deformation (mm)	Max Stress in Screw (von Mises) MPa	Max Stress in Bone (von Mises) MPa	Total Deformation (mm)
**APCS**	479.84	147.19	6.048	569.72	252.91	7.29	405.27	196.12	5.21
**RPCS**	429.31	233.22	4.49	501.13	291.81	5.99	357.94	190.02	3.69
**MS**	335.41	113.16	0.657	458.71	116.81	4.69	278.36	129.75	0.93
**aBCS**	353.78	141.12	1.178	388.48	176.92	3.06	295.95	116.17	0.95
**pBCS**	319.97	126.66	1.033	428.66	90.37	1.41	268.58	111.47	0.86

*APCS: *antegrade posterior column screw*, ***RPCS***: *retrograde posterior column screw*, ***MS***: *magic screw*, ***aBCS***: *anterior both column screw*, ***pBCS***: *posterior both column screw*

Quantitative findings related to fracture surface behavior—including the parameters “Gap in Fracture Surfaces” and “Sliding Distance in Fracture”—across five different internal fixation models under the same three loading conditions are presented in detail in Table 4.

**Table 4:** Fracture surface metrics for different screw fixation methods

	Level walking		Stairs Up		Stairs Down	
Screw Type	Gap in Fracture Surfaces (mm)	Sliding Distance in Fracture Surface (mm)	Gap in Fracture Surfaces (mm)	Sliding Distance in Fracture Surface (mm)	Gap in Fracture Surfaces (mm)	Sliding Distance in Fracture Surface (mm)
**APCS**	0.991	2.043	0.991	2.097	0.991	2.45
**RPCS**	0.998	2.328	1.112	2.072	0.842	1.945
**MS**	0.183	0.646	0.736	1.58	0.232	0.83
**aBCS**	0.312	1.109	0.717	1.563	0.25	0.91
**pBCS**	0.277	0.976	0.641	1.46	0.229	0.738

*APCS: *antegrade posterior column screw*, ***RPCS***: *retrograde posterior column screw*, ***MS***: *magic screw*, ***aBCS***: *anterior both column screw*, ***pBCS***: *posterior both column screw*

**Fig. 4:**
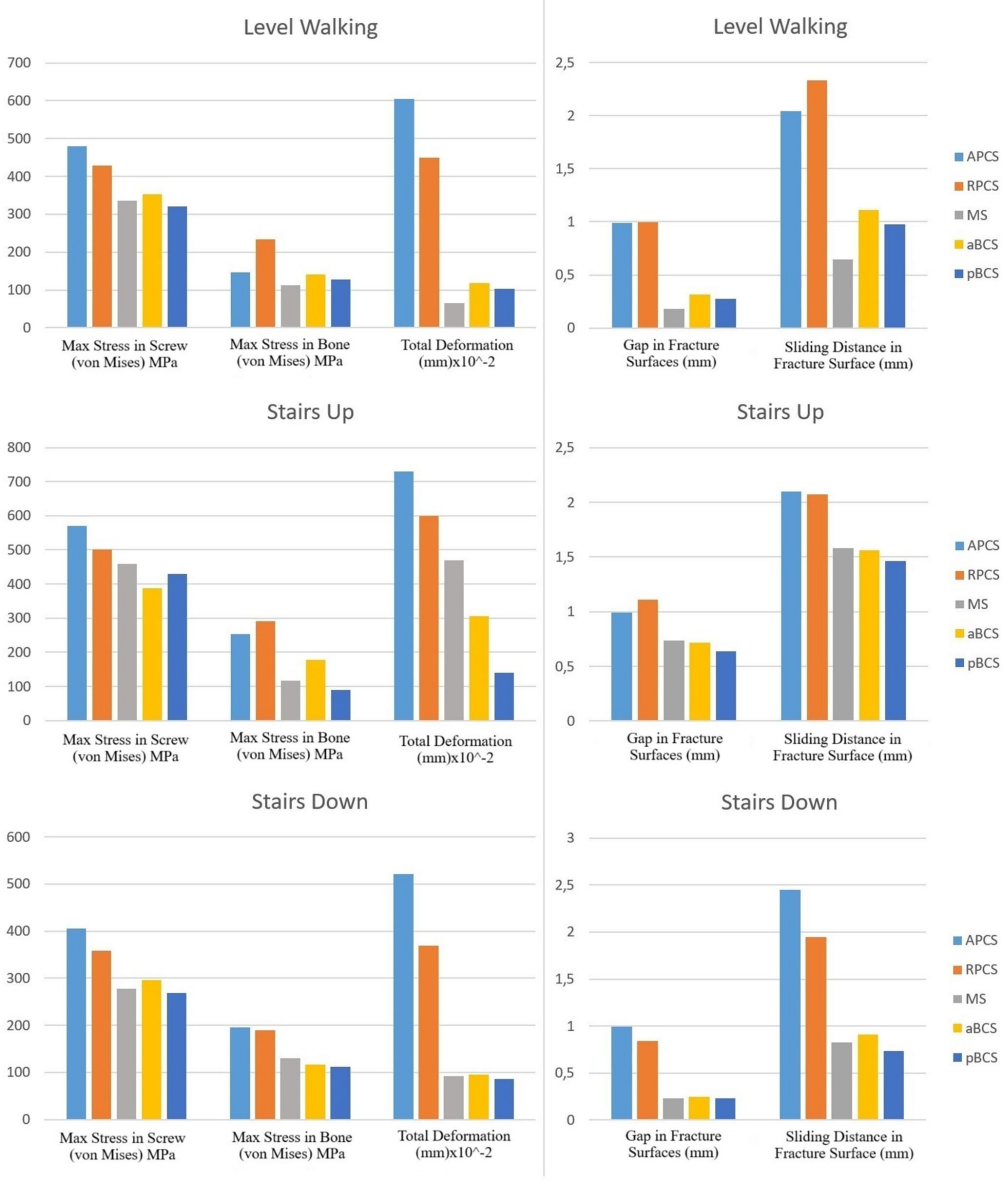
Comparative graphical representation of all biomechanical outcome measures across five screw fixation models and three loading conditions

A complete graphical representation of all biomechanical output parameters—including stress values, deformation, and fracture surface displacement for each screw configuration—is provided in Fig. 4, which illustrates the distribution of these variables across the different fixation strategies and loading scenarios.

In summary, finite element analysis demonstrated that the BCS techniques (aBCS and pBCS) generally exhibited lower screw and bone stress compared to conventional posterior column screw fixation techniques under all loading conditions. Among all loading scenarios, the highest screw stress was observed in the APCS model (569.72 MPa), while the lowest screw stress was recorded in the pBCS model (268.58 MPa). The highest bone stress was found in the RPCS model (291.81 MPa), whereas the lowest bone stress was in the pBCS model (90.37 MPa). In terms of total deformation, the highest value was observed in the APCS model (7.29 mm), while the lowest was found in the MS model (0.657 mm). Regarding fracture surface gap and sliding distance, the highest values were recorded in the APCS model (0.991 mm and 2.45 mm, respectively), whereas the lowest values were measured in the pBCS model (0.229 mm and 0.738 mm, respectively). Across all loading conditions, the pBCS model consistently demonstrated the most favourable biomechanical profile.

## Discussion

Since acetabular column fracture fixation with lag screws was first described by Judet and Letournel in the 1960s, it has been increasingly preferred due to its advantages such as requiring less surgical exposure [30, 31]. The gold standard treatment for displaced acetabular fractures is open reduction and internal fixation (ORIF) [16]. However, percutaneous lag screw fixation is a minimally invasive option that can be applied in the treatment of non-displaced or minimally displaced acetabular column fractures [11, 14, 31, 32]. Furthermore, in acetabular fractures involving both columns, it is a common practice to first perform open reduction and internal fixation for one column, followed by reduction and lag screw fixation of the other column through the same surgical approach [14, 33, 34].

Numerous biomechanical studies have demonstrated that lag screw fixation of acetabular column fractures can provide stability comparable to that of plate-and-screw constructs [35–38].

Various lag screw fixation techniques are available for the treatment of posterior column fractures of the acetabulum. The most commonly known is the antegrade posterior column screw, originally described by Judet and Letournel, which is inserted from the iliac fossa toward the posterior column [16]. Starr et al. later described a retrograde technique in which the screw is inserted percutaneously through the ischial tuberosity [32]. In 2001, Starr et al. also introduced another percutaneous technique involving the quadrilateral surface and posterior column, which they termed the “magic screw” [11]. More recently, Öztürk et al. described a new percutaneous fixation technique for posterior column fractures, termed the “Both Column Screw (BCS)” fixation technique [14, 15].

Numerous in vitro biomechanical studies and finite element analyses have compared the mechanical stability of different internal fixation models for various types of acetabular fractures. For instance, Şibar et al. conducted an in vitro biomechanical study comparing the stability of conventional cannulated screws, talon screws, and traditional plate-screw constructs for the fixation of acetabular posterior column fractures [36]. Wang et al. analyzed the efficacy of infra-acetabular screws (IS) in the fixation of posterior column fractures by comparing APCS, IS, traditional plate-screw techniques, and their combinations [38]. Similarly, Hinz et al. performed a biomechanical comparison of APCS and IS used as adjuncts to plate fixation in acetabular fractures involving the posterior column [39]. These examples reflect a growing body of literature evaluating and comparing the biomechanical performance of various implants and fixation techniques in acetabular fracture management [39–41].

However, there is no comprehensive study directly comparing lag screw techniques for the fixation of acetabular posterior column fractures. In our literature review, we found no studies other than the one by Zhang et al., which was published in Chinese, comparing lag screw fixation methods for acetabular posterior column fractures. Their study compared the Magic Screw, retrograde posterior column screw, single plate, and double plate fixation in the treatment of posterior column fractures [35].

To the best of our knowledge, this is the first biomechanical study to compare lag screw fixation techniques for acetabular posterior column fractures, encompassing both conventional methods such as APCS and RPCS, and innovative approaches such as the Magic Screw (MS) and Both Column Screw (BCS) techniques.

In this study, five different screw fixation techniques were compared based on five distinct biomechanical parameters under three common loading scenarios encountered in daily life: level walking, stair ascent, and stair descent.

Among these parameters, maximum stress within the screw (von Mises stress) reflects the magnitude of mechanical load exerted on the implant. High intramedullary screw stress increases the risk of approaching the elastic limit, potentially leading to implant fatigue or failure over time. In this context, the findings of our study demonstrated that the pBCS configuration exhibited the lowest intramedullary screw stress values across all loading scenarios. Similarly, the aBCS and MS models also showed comparably low stress levels. In contrast, the APCS model consistently recorded the highest screw stress under all loading conditions. These results suggest that the BCS configurations (aBCS and pBCS), as well as the Magic Screw, may offer a biomechanical advantage over conventional APCS and RPCS techniques in terms of long-term screw integrity.

Considering that the APCS and RPCS models exhibited similar biomechanical characteristics in our study, our findings—consistent with those of Zhang et al.—indicate that the Magic Screw demonstrates superior biomechanical performance compared to conventional screw fixation techniques (APCS and RPCS) [35].

Maximum stress within the bone represents the strain occurring in the cortical and cancellous bone surrounding the implant. Elevated bone stress may lead to increased micromotion at the implant–bone interface, cortical damage, or bone resorption. In our study, the lowest bone stress was observed in the MS configuration during level walking, while the pBCS configuration showed the lowest values during stair ascent and descent. Conversely, the highest bone stress was recorded in the RPCS model, followed by the APCS. These findings suggest that reducing peri-implant bone stress is critical for long-term stability, particularly in patients with osteoporotic bone structure. In this regard, the pBCS, aBCS, and MS configurations may offer biomechanical advantages over APCS and RPCS by potentially lowering the risk of bone resorption and implant loosening.

Total deformation reflects the structural change of the system under load and provides an indication of overall rigidity. Lower deformation values suggest that the fixation construct maintains its integrity and exhibits greater biomechanical stiffness. In this study, the lowest deformation was observed in the pBCS configuration under stair ascent and descent conditions, and in the MS model during level walking. A low total deformation value may indicate a level of stability that permits early weight-bearing, which could be particularly advantageous in elderly patients or those with concomitant injuries where early mobilization is critical. In contrast, the highest deformation values were observed in the APCS and RPCS models, indicating comparatively inferior performance in terms of construct rigidity.

Fracture gap and sliding distance are two key parameters that reflect micromotion between fracture fragments and indicate the degree of stability provided by the implant under load. In this study, the MS configuration exhibited the lowest values for both parameters during level walking, while the pBCS configuration showed the lowest values during stair ascent and descent. Similarly, the aBCS model also demonstrated low gap and sliding values. These findings suggest that the MS and BCS (aBCS and pBCS) screw techniques may provide superior fracture stability by minimizing interfragmentary motion. In contrast, the highest gap and sliding distances were observed in the APCS model, followed by the RPCS model. These results indicate that the APCS and RPCS configurations may be more prone to micromotion and therefore represent biomechanically less favourable options for fragment stabilization.

When all parameters are considered together, the pBCS technique consistently demonstrated the most balanced and rigid construct in terms of overall stability. The MS and aBCS models also exhibited similarly high levels of biomechanical performance. In contrast, the conventional APCS and RPCS configurations showed higher stress levels, increased deformation, and greater fracture surface motion under all loading conditions, indicating lower stability and weaker biomechanical profiles.

### Limitations

This study has several limitations. The results obtained from finite element analysis (FEA) may not fully reflect the biological responses following fracture fixation due to individual variability in fracture morphology, technical differences during screw placement, and patient-specific factors such as body mass index (BMI), bone quality, and soft tissue characteristics. Furthermore, the study does not include clinical outcomes. Therefore, the findings should be interpreted as biomechanical guidance for surgical decision-making rather than direct predictors of clinical results.

Additionally, from an axial projection perspective, the posterior column of the acetabulum can be conceptualized as an isosceles triangle with a crescent-shaped base—bounded anteriorly by the acetabular joint, posteriorly by the greater sciatic notch, and medially and laterally by the inner and outer cortices of the column [14]. Accordingly, the aBCS is placed near the base of this triangle (adjacent to the acetabular surface), while the pBCS is positioned toward the apex near the greater sciatic notch. As the triangle narrows toward the apex, anatomical variations—particularly in the female pelvis, where the posterior column tends to be narrower and thinner—may limit the feasibility of placing a 6.5 mm diameter pBCS screw [42].

Therefore, caution is warranted when generalizing these findings, particularly in individuals where the posterior column tends to be narrower—most notably in female patients. In such cases, meticulous preoperative planning, preferably using advanced planning tools or software, is recommended to ensure safe screw placement. This anatomical perspective further underscores the necessity for future feasibility studies aimed at evaluating the applicability of the pBCS technique across diverse pelvic morphologies.

## Conclusion

This study provides a comprehensive finite element-based biomechanical evaluation of the Both Column Screw (BCS) fixation technique in comparison with conventional posterior column screw fixation methods. The findings demonstrate that the BCS configurations (aBCS and pBCS) exhibit a generally superior biomechanical profile in terms of stress distribution, deformation, and fracture surface stability. These results suggest that the BCS technique may offer significant biomechanical advantages over traditional methods and may have the potential to serve as a novel strategy in the surgical management of acetabular posterior column fractures. However, further high-quality, multicenter clinical studies are warranted to validate its clinical efficacy and practical applicability.

## Data Availability

No datasets were generated or analysed during the current study.
